# Development of an immune‐related prognostic model for pediatric acute lymphoblastic leukemia patients

**DOI:** 10.1002/mgg3.1404

**Published:** 2020-07-15

**Authors:** Xi Quan, Nan Zhang, Ying Chen, Hanqing Zeng, Jianchuan Deng

**Affiliations:** ^1^ Department of Hematology The Second Affiliated Hospital Chongqing Medical University Chongqing P.R. China

**Keywords:** immune‐related genes, pediatric acute lymphoblastic leukemia, prognosis, survival analysis

## Abstract

**Background:**

Acute lymphoblastic leukemia (ALL) is the most common hematological malignancy in pediatrics, and immune‐related genes (IRGs) play crucial role in its development. Our study aimed to identify prognostic immune biomarkers of pediatric ALL and construct a risk assessment model.

**Methods:**

Pediatric ALL patients’ gene expression data were downloaded from Therapeutically Applicable Research to Generate Effective Treatments (TARGET) database. We screened differentially expressed IRGs (DEIRGs) between the relapse and non‐relapse groups. Cox regression analysis was used to identify optimal prognostic genes, then, a risk model was constructed, and its accuracy was verified in different cohorts.

**Results:**

We screened 130 DEIRGs from 251 pediatric ALL samples. The top three pathways that DEIRGs may influence tumor progression are NABA matrisome‐associated, chemotaxis, and antimicrobial humoral response. A set of 84 prognostic DEIRGs was identified by using univariate Cox analysis. Then, Lasso regression and multivariate Cox regression analysis screened four optimal genes (*PRDX2*, *S100A10*, *RORB,* and *SDC1*), which were used to construct the prognostic risk model. The risk score was calculated and the survival analysis results showed that high‐risk score was associated with poor overall survival (OS) (*p* = 3.195 × 10^−7^). The time‐dependent survival receiver operating characteristic curves showed good prediction accuracy (Area Under Curves for 3‐year, 5‐year OS were 0.892 and 0.89, respectively). And the predictive performance of our risk model was successfully verified in testing cohort and entire cohort.

**Conclusions:**

Our prognostic risk model can effectively divide pediatric ALL patients into high‐risk and low‐risk groups, which may help predict clinical prognosis and optimize individualized treatment.

## Graphical abstract

Differentially expressed immune‐related genes between the relapse and non‐relapse groups of 251 pediatric acute lymphoblastic leukemia patients were analyzed. Four optimal genes (PRDX2, S100A10, RORB and SDC1) were screened, and then an immune‐related prognostic model based on these four genes were constructed.

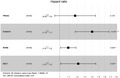

## 1 INTRODUCTION

Acute lymphoblastic leukemia (ALL) is the most frequent malignancy and the leading cause of cancer‐related deaths in pediatrics. The cure rate has exceeded 80% in last decade, owing to improved supportive care and optimized treatment regimens (Brassesco et al., 2018; Kato & Manabe, 2018). However, a significant number of patients still suffer from drug resistance or relapse (Tasian & Hunger, 2017), resulting in treatment failure. In addition, treatment may have to be discontinued because of its high toxicity (Santiago, Vairy, Sinnett, Krajinovic, & Bittencourt, 2017). Taking these factors into account, new biomarkers and precise treatment regimens will be a priority for these patients.

A large number of studies have focused on the development and application of biomarkers in ALL. For example, some researchers have suggested a tumor suppressor role of TLE1 in T‐ALL (Brassesco et al., 2018). In addition, mTOR inhibitors have been used in combination with chemotherapy regiments for the treatment of relapse ALL (Santiago et al., 2017), and bcl‐2 inhibitors have also been used in the treatment for all subtypes of pediatric ALL (Jones et al., 2016). Recent studies have shown that gene expression in patients with recurrent leukemia after transplantation is highly enriched in immune‐related processes (Toffalori et al., 2019). It has also been mentioned that this is related to the escape of tumor cells from the control of allogeneic immune response (Zeiser & Vago, 2019). These results suggest that immune‐related biomarkers may be significant signatures for predicting the prognosis of ALL.

With the development of bioinformatics, the immune‐related genes (IRGs)‐based prognostic signatures have been developed in patients diagnosed with renal papillary cell carcinoma (Wang et al., 2019), colorectal cancer (Bai, Zhang, Xiang, Zhong, & Xiong, 2020), and lung adenocarcinoma (Song et al., 2019), which can predict survival outcomes. However, the prognostic value of IRGs‐based signatures in pediatric ALL patients is still unknown.

The purpose of this study was to investigate the clinical significance of IRGs on the prognosis of pediatric ALL and its biological function. In this paper, we comprehensively analyzed the expression profile data and the clinical information of pediatric ALL patients. A prognostic model based on IRGs was developed and validated in public dataset, which may be helpful in predicting prognosis and optimizing individualized treatment.

## 2 MATERIALS AND METHODS

### 2.1 Gene expression datasets

The transcriptomic data and corresponding clinical information of 251 pediatric ALL patients were downloaded from the Therapeutically Applicable Research to Generate Effective Treatments (TARGET) portal (https://ocg.cancer.gov/programs/target) (Kang et al., 2010). And the 2,498 IRG sets were obtained from the ImmPort database (https://www.immport.org/home) (Bhattacharya et al., 2014). The expression data were preprocessed by the following steps: (a) removing samples with no clinical data; (b) removing samples that expression data and clinical information did not match; (c) preserving only the expression profiles of IRGs. Form this, 185 patients with complete gene expression profiles and clinical information were utilized to further analyze the model. The data downloaded from the TARGET and ImmPort databases is publicly available and accessible, therefore, this study does not require ethics committee approval.

### 2.2 Identification of DEIRGs

The pediatric ALL samples were divided into relapse group and non‐relapse group. And the treatment regimens before the study endpoint events were chemotherapy treatments. The differentially expressed IRGs (DEIRGs) were screened by using edge R package (Robinson, McCarthy, & Smyth, 2010) in R3.6.2 software. The FDR < 0.05 and |log2 fold‐change [FC]| > 1.5 were cutoff values. Then, the gene expression values were visualized by pheatmap package (Li, Zhang, Rui, Sun, & Guo, 2018). Enrichment analysis was performed to predict the biological functions of the DEIRGs by using Metascape (http://metascape.org/), an online bioinformatics pipeline (Zhou et al., 2019).

### 2.3 Construction of the risk score prognostic model

The 185 samples were randomly divided into a training cohort (*n* = 93) and a testing cohort (*n* = 92). The training cohort was used to build the risk score prognostic model, the testing and entire TARGET cohorts were used to test the model. First, univariate Cox analysis was used to identify possible prognostic DEIRGs (PDEIRGs), and *p* < .05 was considered significant. Then, the Lasso regression was applied to select potential risk genes and eliminate genes that would overfit the model. Finally, we used multivariate Cox regression analysis to construct a prognostic risk model.

### 2.4 Risk score calculation and Model validation

To evaluate the contribution of each gene to prognosis, the multivariate Cox regression analysis was performed. Then, we obtained a computational formula that weight the expression values of selected genes with the regression coefficients as follows:

$$\text{Risk score}\left(\text{patient}\right)=\sum_{i=1}^{n}\text{coefficient}\left(\text{gene}\;i\right)\text{expression value of}\left(\text{gene}\;i\right)$$

The risk model was used to measure the prognostic risk of each pediatric ALL patient.

We substituted the expression profile data into the model to calculate the risk score of each sample from the training cohort and entire TARGET cohort. Then, Kaplan–Meier survival analysis, receiver operating characteristic (ROC) analysis, risk score distribution, survival status, and risk gene expression of the training cohort, entire TARGET cohort were performed to verify our risk score prognostic model. Multivariate Cox regression analysis was used to assess the independent prognostic ability of the model.

### 2.5 Statistical analyses

Statistical analyses were performed using R software (https://www.r‐project.org/) and Perl (https://www.activestate.com/products/perl/). Univariate Cox regression analysis was used to identify factors affecting the survival of pediatric ALL patients. Lasso regression was used to evaluate univariate analysis of the link between PDEIRGS. Multivariate Cox regression analysis was used to identify prognostic factors. Kaplan–Meier curves and log‐rank tests were used to analyze the survival data. An Area Under Curve (AUC) > 0.60 was regarded as acceptable for predictions, and an AUC > 0.75 was deemed to have excellent predictive value (Cho et al., 2019; Han et al., 2018).

## 3 RESULTS

### 3.1 DEIRGs screening based on the pediatric ALL samples

**Figure 1:**
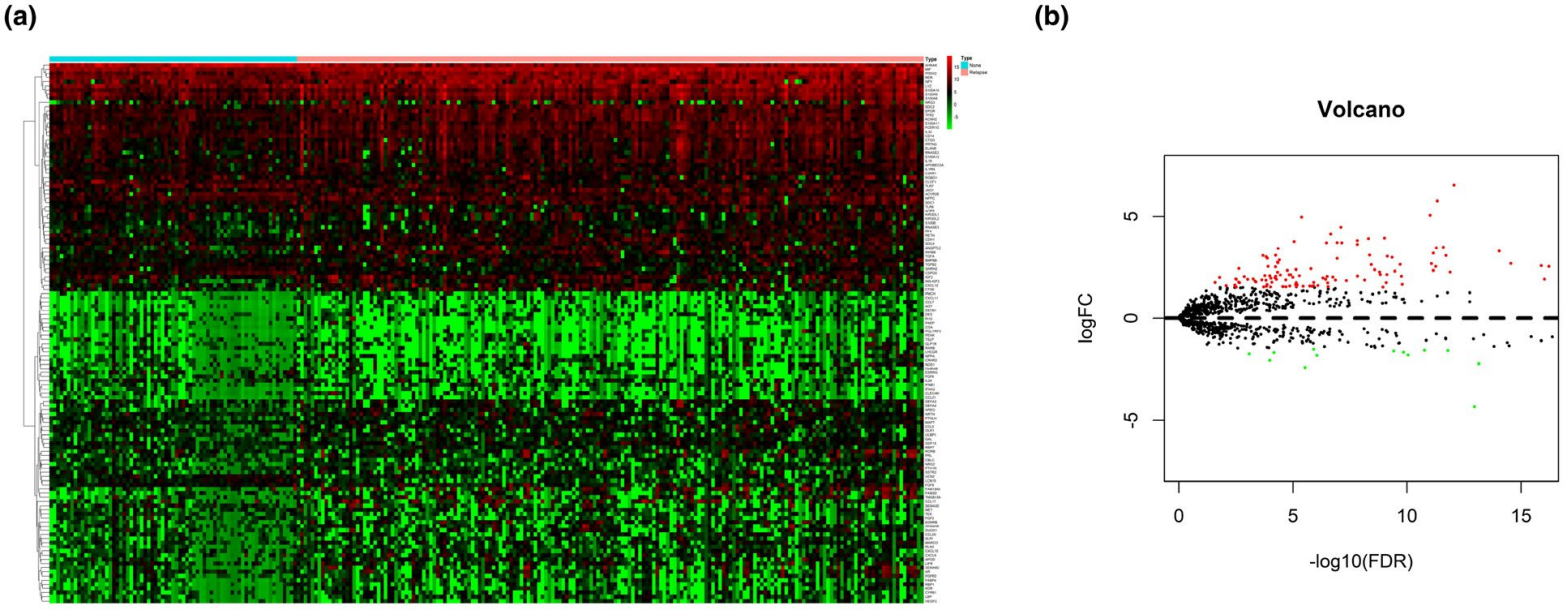
Differentially expressed immune‐related genes. (a) Heatmap of DEIRGs; the green spectrum means low gene expression while the red means high gene expression. (b) Volcano plot of DEIRGs; the green dots indicate downregulated IRGs, the red dots indicate upregulated IRGs, and the black dots represent IRGs that were not significantly differentially expressed. DEIRGs, differentially expressed IRGs; IRGs, immune‐related genes

The mRNA expression data of 2,498 IRGs in pediatric ALL (*n* = 251) from TARGET database was examined. After screening the expression data by using edge R package, a total of 130 DEIRGs were obtained from with relapse group (*n* = 180) and without group (*n* = 71). The results showed that 10.8% (14/130) of DEIRGs were downregulated in relapse group while 89.2% (116/130) of DEIRGs was significantly upregulated (FDR < 0.05, |log2 fold‐change [FC]| > 1.5) (Figure 1a,b).

**Figure 2:**
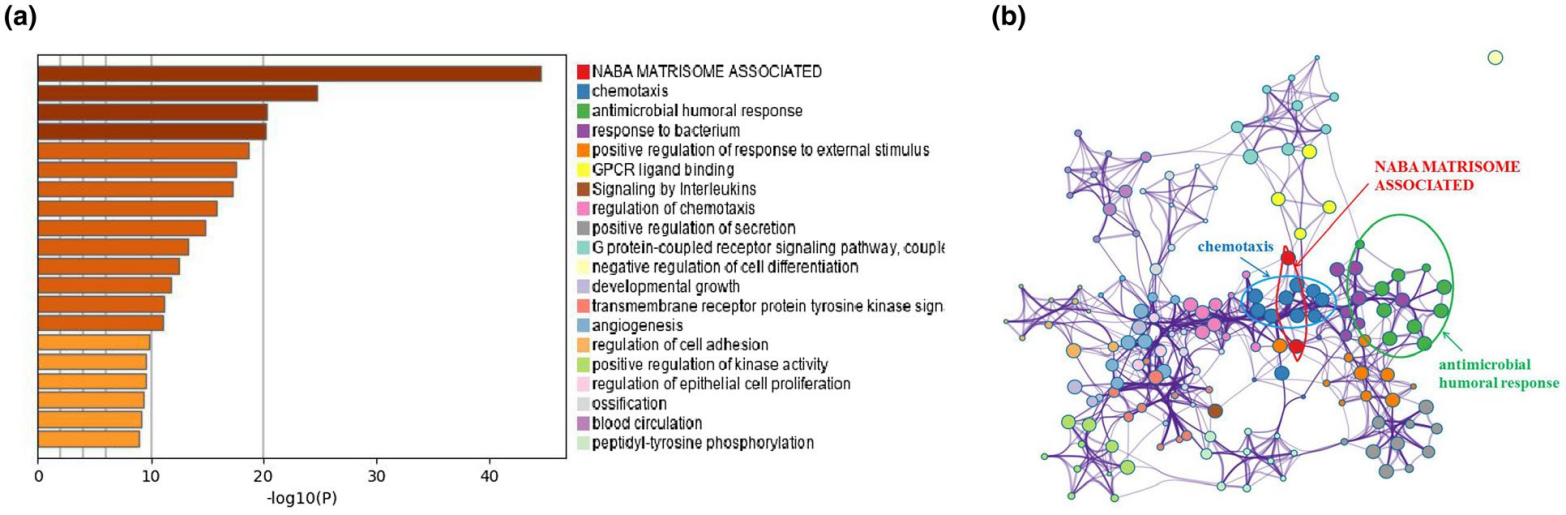
Biological functions of DEIRGs. (a) Significantly enriched pathways of the DEIRGs. (b) Network of enriched pathways. Each node represents an enriched GO term and node size represents the number of gene in the pathways. DEIRGs, differentially expressed IRGs

Then, we conducted enrichment analysis to identify the possible biological functions of DEIRGs. Data showed that the top three signaling pathways affected by DEIRGs were NABA matrisome‐associated, chemotaxis, and antimicrobial humoral response [Figure 2a,b]. All these three signaling pathways were reported to be associated with tumor progression (Chen, Lin, Wu, Her, & Hui, 2009; Naba et al., 2012; Shields et al., 2007), providing evidence for further study on the mechanism of pediatric ALL progression.

### 3.2 Identification of prognostic DEIRGs

The 185 samples were randomly divided into a training cohort (*n* = 93) and a testing cohort (*n* = 92), see Table 1. To identify possible prognostic DEIRGs, we performed a univariate Cox regression analysis of the expression of each DEIRG in the training cohort. As a result, 84 PDEIRGs were found to be significantly associated with the overall survival (OS) of pediatric ALL patients (*p* < .05).

**Table 1:** Clinical information of pediatric ALL patients in the training and validation cohorts

	Training cohort (*n* = 93)	Testing cohort (*n* = 92)	Entire TARGET cohort (*n* = 185)
Sex			
Male	47 (50.5%)	44 (47.8%)	91 (49.2%)
Female	46 (49.5%)	48 (52.2%)	94 (50.8%)
Age at diag (years)			
<10	64 (68.8%)	60 (65.2%)	124 (67%)
≥10	29 (31.2%)	32 (34.8%)	61 (33%)
WBC at diag (×10^9^/L)			
<50	60 (64.5%)	57 (62%)	117 (63.2%)
≥50	33 (35.5%)	35 (38%)	68 (36.8%)
CNS status at diag			
CNS1	73 (78.5%)	76 (82.6%)	149 (80.5%)
CNS2	19 (20.4%)	14 (15.2%)	33 (17.8%)
CNS3	1 (1.1%)	2 (2.2%)	3 (1.7%)
First event			
Relapse	57 (61.3%)	64 (69.6%)	121 (65.4%)
None	36 (38.7%)	28 (30.4%)	64 (34.6%)
Vital status			
Dead	39 (41.9%)	43 (46.7%)	82 (44.3%)
Alive	54 (58.1%)	49 (53.3%)	103 (55.7%)

Abbreviations: ALL, acute lymphoblastic leukemia; CNS, central nervous system; CNS1: no lymphoblasts in CSF; CNS2: present lymphoblasts in CSF, WBC count of the CSF < 5 cells/µl; CNS3: present lymphoblasts in CSF or a cranial nerve palsy, WBC count of the CSF ≥ 5 cells/µl; diag, diagnosis; TARGET, Therapeutically Applicable Research to Generate Effective Treatments; WBC, white blood cell count.

### 3.3 Screening prognostic genes for constructing risk model

**Figure 3:**
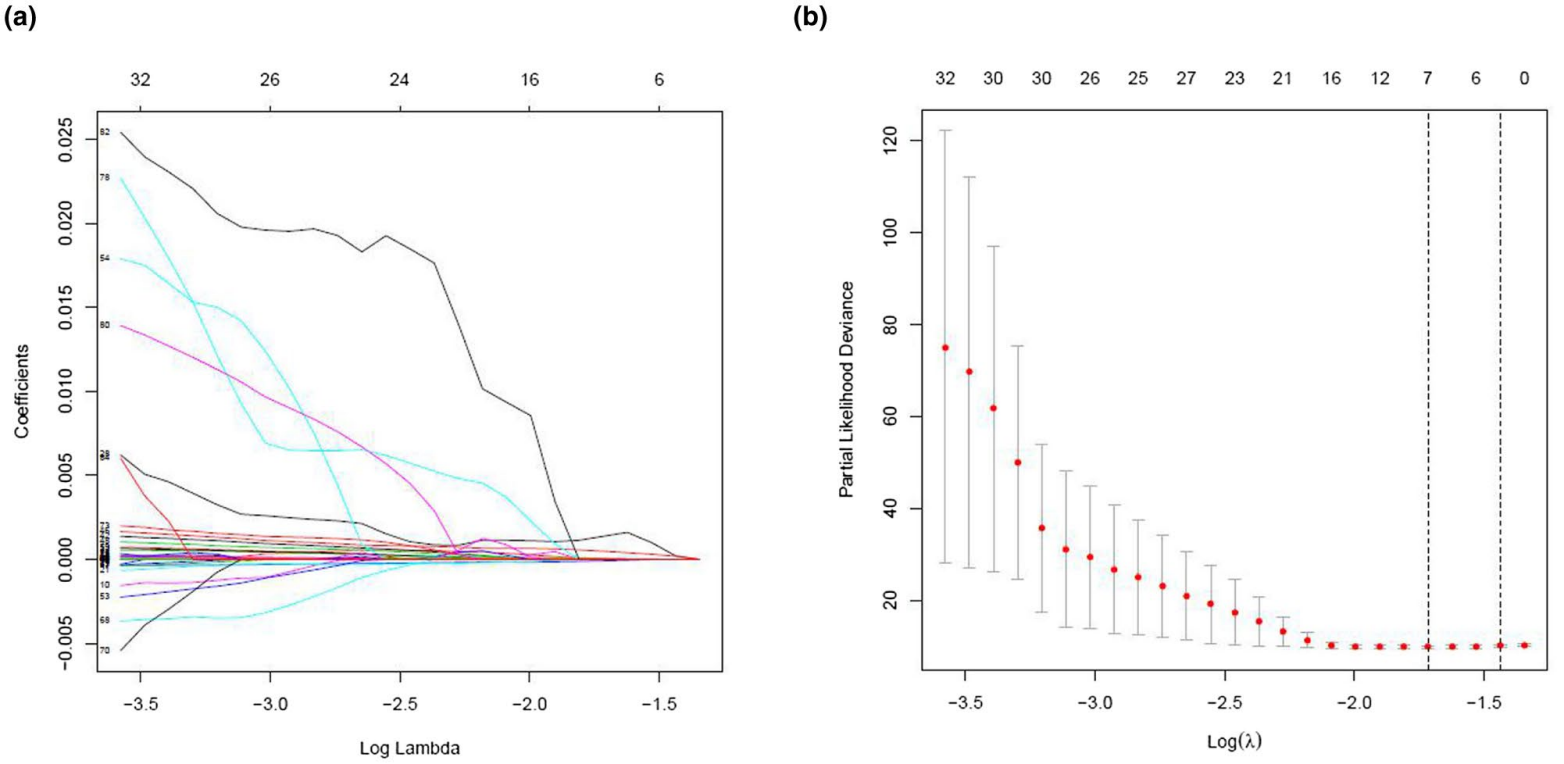
(a and b) PDEIRGs screened through Lasso regression

**Figure 4:**
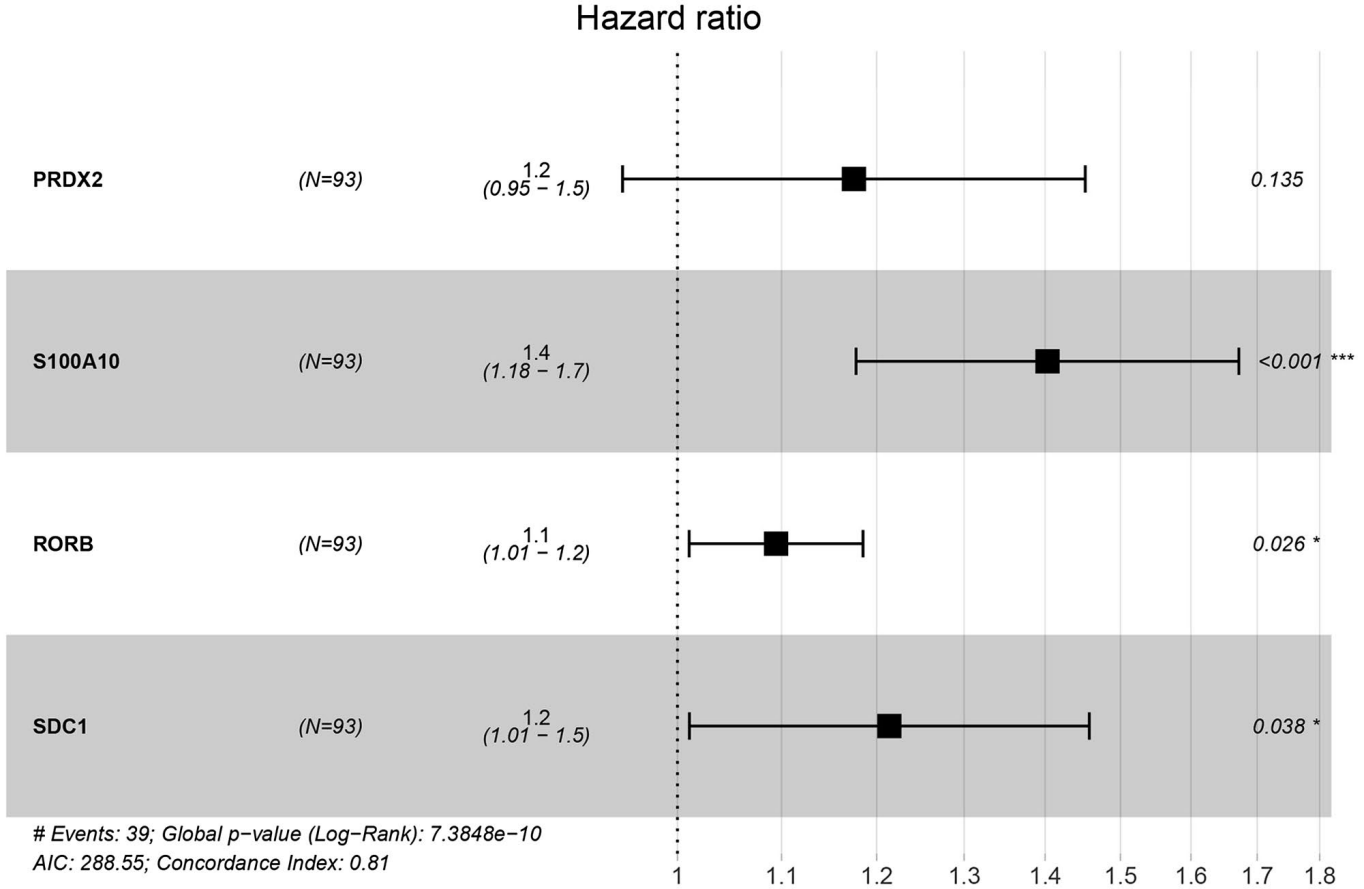
Risk genes of the prognostic risk model. *, *p* < .05; ***, *p* < .001

We further analyzed and screened PDEIRGs for constructing cox regression hazard model. First, to avoid model overfitting, we used Lasso regression to remove PDEIRGs that are highly correlated to each other. Therefore, we obtained seven candidate PDEIRGs (Figure 3a,b). Then, multivariate Cox proportional risk regression analysis was performed (with forward selection and backward selection). Finally, we obtained four optimal PDEIRGs (risk genes) to incorporate into the prognostic risk model: *PRDX2*, *S100A10*, *RORB,* and *SDC1*. These four genes were identified as high‐risk genes (predicting a poor prognosis) in terms of the OS of patients (Figure 4).

### 3.4 Construction of prognostic risk model in training cohort

Based on the results of multivariate Cox regression analysis, we constructed a model to assess the significance of risk genes in predicting prognosis in pediatric ALL patients. The computational formula was as follows:

$$\text{Training cohort risk score}=\left(0.1615\times \text{expression of PRDX}2\right)+\left(0.3387\times \text{expression of S}100A10\right)+\left(0.0903\times \text{expression of RORB}\right)+\left(0.1940\times \text{expression of SDC}1\right).$$

**Figure 5:**
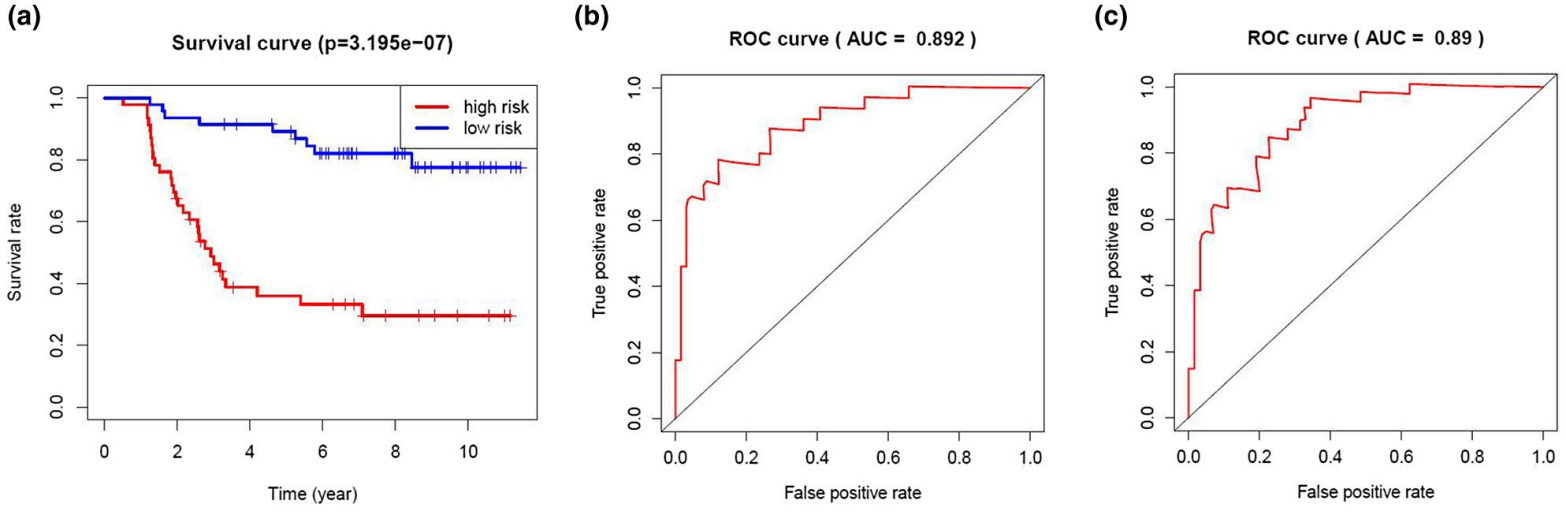
Prognosis analysis of training cohort. (a) Kaplan–Meier curve analysis of the high‐risk and low‐risk groups. Time‐dependent ROC curve analysis for the predictive accuracy of the risk model for 3‐year (b) and 5‐year OS (c). OS, overall survival; ROC, receiver operating characteristic

**Figure 6:**
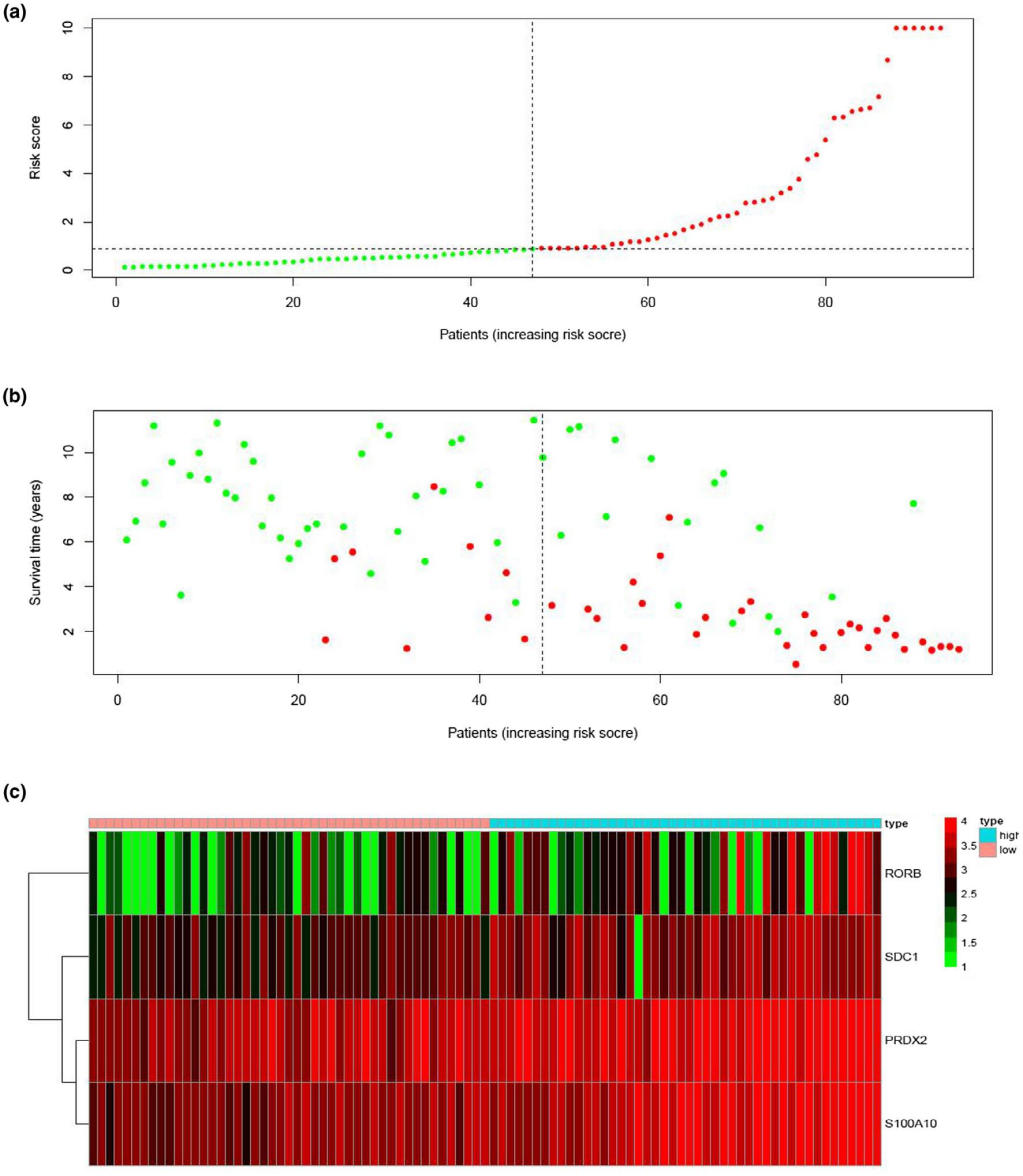
Prognosis analysis of training cohort. (a) Risk score distribution of patients based on the prognostic risk model. (b) Survival status of patients in different groups. (c) Heatmap of expression profiles of risk genes

We calculated the risk score of each patient in the training cohort using the risk model, and patients were sorted into a high‐risk group (*n* = 46) and a low‐risk group (*n* = 47). To investigate the difference in prognosis between the high‐risk and low‐risk groups, we created a Kaplan–Meier curve based on the log‐rank test. The prognosis was better in the low‐risk group than in the high‐risk group (*p* = 3.195 × 10^–7^) (Figure 5a). The OS rates at 3 years and 5 years for the high‐risk group in the training cohort were 46.3% and 33.2%, respectively, while the corresponding rates for the low‐risk group were 91.5% and 86.9%, respectively. Then, we tested the predictive accuracy of the model for 3‐year and 5‐year OS through the time‐dependent ROC curves. The AUC values for the prognostic model were 0.892 at 3 years and 0.89 at 5 years (Figure 5b,c). We then sorted and analyzed the distribution of patients’ risk scores in the training cohort (Figure 6a). The survival status of each patient in the training cohort is marked on the dot plot in Figure 6b. The heatmap we completed showed the expression of risk genes in both risk groups (Figure 6c). In the high‐risk group of the training cohort, four high‐risk genes (*PRDX2*, *S100A10*, *RORB,* and *SDC1*) were upregulated. In the low‐risk group, the expression of these risk genes was downregulated.

### 3.5 Verification of the performance of the prognostic model

To validate the predictive ability of the prognostic risk model, we used it to analyze the testing cohort (the remaining 92 patients from the 185 total) and the entire TARGET cohort. First, the risk score for each patient in the testing cohort and the entire TARGET cohort was calculated according to the coefficient value of the four risk genes. Patients were divided into high‐risk and low‐risk groups with the median risk score of the training cohort utilized as the cutoff value. In the testing cohort, 52 patients were divided as high risk and 40 were divided as low risk. In the entire TARGET cohort, 98 patients were classified as high risk and 87 were classified as low risk.

**Figure 7:**
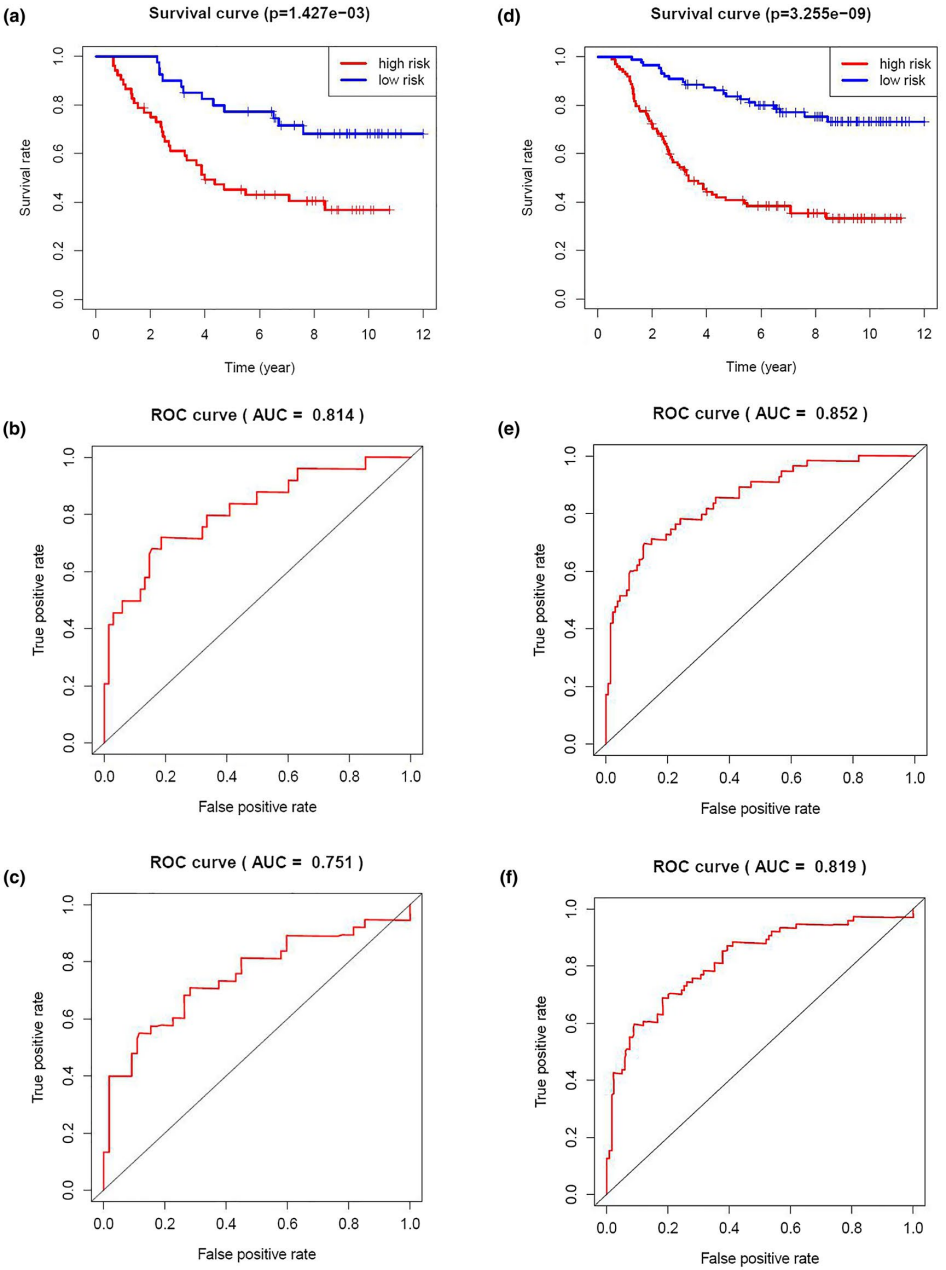
Prognosis analysis of testing cohort and entire TARGET cohort. Kaplan–Meier curve analysis of the high‐risk and low‐risk groups ((a) for testing cohort, (d) for entire TARGET cohort). Time‐dependent ROC curve analysis for the predictive accuracy of the risk model for 3‐year ((b) for testing cohort, (e) for entire TARGET cohort) and 5‐year OS ((c) for testing cohort, (f) for entire TARGET cohort). ROC, receiver operating characteristic; OS, overall survival; TARGET, Therapeutically Applicable Research to Generate Effective Treatments

Then, Kaplan–Meier survival analysis was performed for both the testing cohort and the entire TARGET cohort. Patients of high risk were with poor OS compared with those of low risk in both the testing cohort (*p* = 1.427 × 10^–3^) and the entire TARGET cohort (*p* = 3.255 × 10^–9^) (Figure 7a,d). In the testing cohort, the OS rates at 3 years and 5 years for the high‐risk group were 59.1% and 45.2%, respectively, while the corresponding rates for the low‐risk group were 87.5% and 77.3%, respectively. In the entire TARGET cohort, the OS rates at 3 years and 5 years for the high‐risk group were 54.3% and 40.7%, respectively, while the corresponding rates for the low‐risk group were 89.7% and 82.5%, respectively. To evaluate the accuracy in prognosis prediction of our four‐gene model, we performed time‐dependent ROC curve analysis. In the testing cohort, the AUCs at 3 and 5 years were 0.814 and 0.751, respectively (Figure 7b,c). In the entire TARGET cohort, the AUCs at 3 and 5 years were 0.852 and 0.819, respectively (Figure 7e,f).

**Figure 8:**
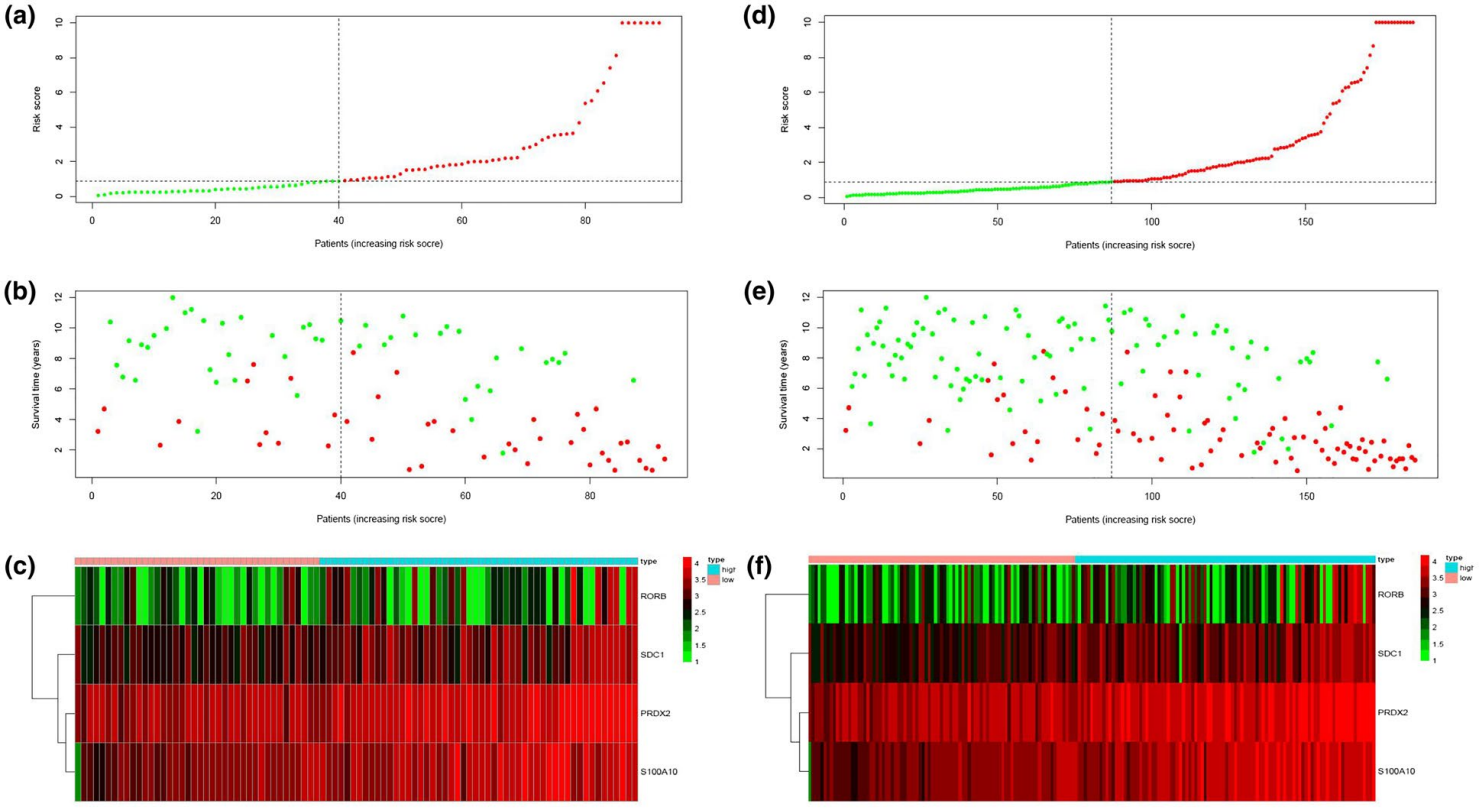
Prognosis analysis of testing cohort and entire TARGET cohort. Risk score distribution of patients based on the prognostic risk model ((a) for testing cohort, (d) for entire TARGET cohort). Survival status of patients in different groups ((b) for testing cohort, (e) for entire TARGET cohort). Heatmap of expression profiles of risk genes ((c) for testing cohort, (f) for entire TARGET cohort). TARGET, Therapeutically Applicable Research to Generate Effective Treatments

The risk score distribution, survival status, and risk gene expression in the testing cohort and the entire TARGET cohort are shown in Figure 8a–f. Similar to the results in the training cohort, risk gene levels were lower in the low‐risk group than in the high‐risk group. These results suggested that our prognostic risk model can accurately predict the prognosis of pediatric ALL patients.

The univariate and multivariate Cox analysis of risk score generated by our model and clinical parameters in entire TARGET cohort is shown in Table 2. The univariate analysis indicated that the variables of age, minimal residual disease (MRD) status at day 29 of induction therapy, and risk score were associated with the prognosis of pediatric ALL patients. And in the multivariate analysis, the risk score can serve as an independent prognostic factor for OS in the entire TARGET cohort (*p* < .05). These results suggested that our prognostic risk model can be independently used to predict the prognosis of pediatric ALL patients. In addition, the variables of age, MRD status at day 29 also had important prognostic value in the multivariate analysis (*p* < .05).

**Table 2:** Univariate and multivariate Cox regression analyses of the entire TARGET cohort

Variables	Univariate analysis		Multivariate analysis	
	HR (95% CI)	*p* value	HR (95% CI)	*p* value
Risk score (from risk model)				
High versus low	1.25 (1.19–1.32)	6.07E‐19	1.29 (1.22–1.36)	4.29E‐20
Age at diagnosis				
≥10 versus <10 years old	2.15 (1.38–3.34)	6.72E‐04	2.24 (1.43–3.51)	4.27E‐04
Gender				
Male versus female	1.40 (0.90–2.18)	0.131	1.69 (1.07–2.65)	2.37E‐02
WBC at diagnosis				
＞50 versus ≤50 × 10^9^/L	0.93 (0.59–1.47)	0.751	0.98 (0.61–1.57)	0.927
CNS status at diagnosis				
CNS3/CNS2 versus CNS1	1.05 (0.60–1.84)	0.871	1.05 (0.59–1.87)	0.87
MRD day 29				
≥10^−4^ versus ＜10^−4^	1.80 (1.15–2.79)	9.46E‐03	2.06 (1.30–3.25)	2.09E‐03

Note

MRD day 29, minimal residual disease status in bone marrow, by flow cytometry, at day 29 of induction therapy.

Abbreviations: CI, confidence interval; CNS, central nervous system; CNS1: no lymphoblasts in CSF; CNS2: present lymphoblasts in CSF, WBC count of the CSF < 5 cells/µl; CNS3: present lymphoblasts in CSF or a cranial nerve palsy, WBC count of the CSF ≥ 5 cells/µl; HR, hazard ratio; MRD, minimal residual disease; TARGET, Therapeutically Applicable Research to Generate Effective Treatments; WBC, white blood cell count.

## 4 DISCUSSION

Although cure rate of pediatric ALL have improved recently, some patients still suffer from relapse and refractory. With the development of second‐generation sequencing, researchers expect to improve clinical outcomes through more accurate risk stratification and molecular‐targeted therapies (Tasian & Hunger, 2017). Studies have shown that gene expression profiles of patients with relapse leukemia are highly enriched in immune‐related processes (Toffalori et al., 2019). In addition, some tumor relapses are associated with cancer cells mimicking the IRGs of healthy cells (van der Bruggen et al., 1991; Knuth, Danowski, Oettgen, & Old, 1984; Old, 1981; Sahin et al., 1995; Schreiber, Old, & Smyth, 2011). Therefore, immune‐related biomarkers may be an important indicator of prognosis in pediatric ALL.

We analyzed the differential immune gene expression between the relapse and non‐relapse groups of 251 pediatric ALL patients and screened 130 DEIRGs. Pathway enrichment analysis was performed to explore the potential biological mechanisms of them. And the top three pathways were NABA matrisome‐associated, chemotaxis, and antimicrobial humoral response, which were reported to be involved in tumor development (Chen et al., 2009; Naba et al., 2012; Shields et al., 2007). Based on comprehensive analysis, we identified four optimal genes (*PRDX2*, *S100A10*, *RORB*, and *SDC1*) and used them to conduct a prognostic risk model for pediatric ALL patients. The model was able to classify pediatric ALL patients into two subgroups with statistically different survival outcomes, which were validated in both the testing cohort and the entire TARGET cohort. In addition, we verified and analyzed the risk score distribution, survival status, and risk gene expression of testing cohort and entire TARGET cohort. We came to the conclusion that low‐risk group had lower levels of the risk gene than high‐risk group, which is similar to that of the training cohort. These results suggest that the model may represent the risk status of pediatric ALL patients and provide reliable prognostic value for them. And the multivariate Cox regression analysis confirmed that our model could independently predict the prognosis of pediatric ALL patients.

We identified four optimal signatures from IRGs: *PRDX2*, *S100A10*, *RORB,* and *SDC1*. *PRDX2* can regulate oxidative and metabolic stress, whose carcinogenic role in several solid cancers has been reported (Kim et al., 2000; Stresing et al., 2013). *PRDX2* has also been shown to induce the growth of lymphoma cells (Trzeciecka et al., 2016). And *S100A10* can promote the invasion and metastasis of cancer by increasing the production of fibrinolytic enzyme (Choi, Fogg, Yoon, & Waisman, 2003; Madureira et al., 2016; O'Connell, Madureira, Berman, Liwski, & Waisman, 2011; Zhang, Fogg, & Waisman, 2004). *RORB* regulates Wnt pathway activity, which may be correlated with tumorigenesis and tumor stages (Wen et al., 2017). In addition, *SDC1* has been reported to play an important role in the malignant progression of tumors (Li et al., 2019). At present, no reports concerning these genes were published in ALL, so the role of them in pediatric ALL needs further investigation.

Many researches focused on the relapse and prognosis of leukemia. Cristina Toffalori et al. found that the gene expression profile of patients with recurrence was highly enriched in immune‐related processes by analyzing the genome of patients with acute myeloid leukemia transplantation, and frequent new genomic changes in patients who relapsed after transplantation were observed (Toffalori et al., 2019), which was consistent with Miguel et al's report (Waterhouse et al., 2011). Joanna et al. suggested that the most striking characteristics were pathways leading to drug‐resistant phenotypes in ALL relapsed patients with high‐resolution genomic techniques, which can be targeted to prevent or treat relapse (Pierro, Hogan, Bhatla, & Carroll, 2017). Plenty of studies on prognosis of leukemia, nevertheless, no immune gene‐related prognostic research of pediatric ALL has been carried out. Therefore, we focused on the pediatric ALL sample data from public dataset TARGET, which includes comprehensive clinical information and sequencing data. We used multiple algorithms (including univariate Cox, multivariate Cox, and Lasso regression) at the genome‐wide level to construct a risk model for predicting the prognosis of pediatric ALL patients. And the model was successfully verified in testing cohort and entire TARGET cohort. Therefore, the research data are comprehensive and research method is reliable. Our predictive model can represent the risk status and provide reliable prognostic value for the whole cohort and subgroups of pediatric ALL patients. Still, our survey has some limitations. We used retrospective data that were not validated in prospective clinical trials. In addition, further studies are needed on the mechanism by which IRGs affect pediatric ALL prognosis.

## 5 CONCLUSIONS

In conclusion, we identified and verified four risk signatures based on IRGs. Then based them a risk model for pediatric ALL patients was developed, which can classify patients into high‐risk and low‐risk groups. These findings may provide insights for predicting clinical outcomes and individualized treatment based on risk scores.

## CONFLICT OF INTERESTS

The authors declare that they have no competing interests.

## AUTHOR CONTRIBUTIONS

XQ and NZ designed the study and downloaded the data. XQ performed the data analyses. XQ, YC, and HZ wrote the manuscript. JD revised the manuscript. All authors read and approved the final manuscript.

## Data Availability

Expression data can be found in the Therapeutically Applicable Research to Generate Effective Treatments (TARGET) portal (https://ocg.cancer.gov/programs/target) and immune‐related gene sets can be accessed from the ImmPort database (https://www.immport.org/home).
